# Global trends in the incidence and mortality of esophageal cancer from 1990 to 2017

**DOI:** 10.1002/cam4.3338

**Published:** 2020-08-04

**Authors:** Jiahui Fan, Zhenqiu Liu, Xianhua Mao, Xin Tong, Tiejun Zhang, Chen Suo, Xingdong Chen

**Affiliations:** ^1^ State Key Laboratory of Genetic Engineering and Collaborative Innovation Center for Genetics and Development School of Life Sciences Fudan University Shanghai China; ^2^ Department of Epidemiology & Ministry of Education Key Laboratory of Public Health Safety School of Public Health Fudan University Shanghai China; ^3^ Fudan University Taizhou Institute of Health Sciences Taizhou China; ^4^ State Key Laboratory of Genetic Engineering Human Phenome Institute Fudan University Shanghai China

**Keywords:** age specificity, correlation, esophageal cancer, genderspecificity, geographic variation, temporal variation

## Abstract

**Background:**

The incidence and mortality of esophageal cancer are high, with 5.90 new cases and 5.48 deaths per 100 000 people worldwide in 2017. The prognosis of esophageal cancer is poor, with an overall 5‐year survival rate of less than 20%. Esophageal cancer in different geographical locations has different etiologies, and the incidence and mortality of esophageal cancer continue to rise in some regions.

**Methods:**

We collected incidence and mortality data by age and gender for 195 countries and territories from 1990 to 2017 in the Global Burden of Disease (GBD) database. And we used these data to calculate the estimated annual percentage change (EAPC) to quantify trends in morbidity and mortality. Then we analyzed the gender‐ and age‐specific incidence and mortality in esophageal cancer to targeted high‐risk populations. Finally, we analyzed the correlation between the age‐standardized mortality rate (ASMR) and both the EAPC and social‐demographic index (SDI), and we calculated the Pearson correlation coefficient.

**Results:**

We found that Malawi, East Asia, and high‐middle SDI regions had the highest age‐standardized incidence rate (ASIR) and ASMR, and the ASIR and ASMR in western Sub‐Saharan Africa showed an upward trend. Our study also showed that the incidence and mortality in esophageal cancer were highest in men and in the 70+ years age group, and they presented a decreasing trend in most regions, but the 15‐49 years age groups in Australasia, Caribbean, and Oceania and the 70+ years age group in High‐Income North America, Oceania and high‐SDI regions presented an increasing trend. There were significant negative associations between ASMR at baseline and EAPC and between ASMR and SDI in 2017.

**Conclusion:**

By analyzing the global distribution of incidence and mortality in esophageal cancer, trends over time, and gender and age specificity, we can understand the heterogeneity of its global trends. This heterogeneity can help us to identify high‐risk groupsand to provide clues for the exploration of the etiology and early prevention of the disease.

## Graphical abstract

Our study explores areas where morbidity and mortality continue to rise, and refines them into populations of different ages and genders. The heterogeneity of the incidence and mortality trends in esophageal cancer means that we can target high‐risk populations and provide clues for the exploration of the etiology and early prevention of the disease.

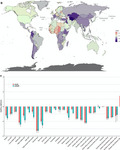

## 1 INTRODUCTION

Among all cancers worldwide, the incidence of esophageal cancer ranks 7th, and the mortality rate ranks 6th.^1^, ^2^ The incidence of esophageal cancer shows significant geographic distribution differences around the world, with particularly high incidence occurring in some regions of South America, Asia, and southern and eastern Africa, where morbidity is 20 times higher thanin some regions of West Africa.^3^, ^4^ The prognosis of esophageal cancer is poor because it usually does not show obvious symptoms in the early stage of the disease, leading to its detection at the later stage of the disease.^5^ At this time, more than half of the patients have distant metastases and some irreversible lesions. This has led to a frustrating overall 5‐year survival rate, although it has been increasing over time, which is still less than 20%.^6^, ^7^, ^8^

Esophageal cancer mainly occurs in developing countries, and its cases and deaths account for more than 80% of all regions.^9^ The high incidence in some regions is a prominent characteristic of this malignant tumor, and in these hot spots of esophageal cancer, esophageal squamous cell carcinoma (ESCC) is the predominant histological subtypes.^10^, ^11^ In recent years, research on esophageal cancer has focused on Asia and Africa. In Asia, the region with a high incidence of esophageal cancer is often referred to as the “esophageal cancer belt”. This belt runs from Northern Iran through Central Asia to Mongolia and North‐Central China with 90% of cases being ESCC.^12^, ^13^ The extension of this “Asian esophageal cancer belt” is consistent with the ancient Silk Road established by China approximately 2000 years ago.^14^ In the African region, ESCC mainly influences the “north‐south corridor”in easterly, which extends from Ethiopia and Kenya to South Africa.^15^, ^16^ The emergence of esophageal cancer in this corridor has been more than half a century old, dating back to 1969.^17^, ^18^ In contrast, the incidence of esophageal cancer is usually lower in Western countries.^19^ In recent years, the incidence of esophageal adenocarcinoma (EAC), another histological subtype of esophageal cancer, has been increasing in some western developed countries and has even exceeded that of ESCC.^20^, ^21^

Etiological clues to esophageal cancer can be gained from previous descriptive studies, including investigations of risk factors such as gender differences.^22^, ^23^, ^24^ Establishing whether there are gender differences in different contexts, that is, exploring whether it may be related to gender‐related behaviors and exposures, can provide clues about the nature of risk factors. In addition, a prominent feature of the esophageal cancer burden in East Africa is the higher number of young patients (aged < 40).^25^ Whether the gender differences in young age groups are significant and at what age the gender differences are manifested, the discussion of these issues has special value in exploring the potential role of early exposures and susceptibility.^26^ Therefore, an exploration of the global incidence and mortality trends and the location of high‐risk populations of esophageal cancer through gender and age stratification are necessary for the exploration of the etiology and early prevention of it.

In this paper, we combined age‐standardized incidence rate (ASIR) and age‐standardized mortality rate (ASMR) data of esophageal cancer in 195 countries and territories, 21 geographic regions and 5 SDI regions, and we analyzed the gender‐specific and age‐specific incidence and mortality data: (a) we systematically summarized the global burden in esophageal cancer; (b) we explored regions where ASIR and ASMR continue to rise; and (c) we identified high‐risk populations through gender and age stratification.

## 2 MATERIALS AND METHODS

### 2.1 Study data

The Global Health Data Exchange (GHDx) query tool (http://ghdx.healthdata.org/gbd‐results‐tool) in the Global Burden of Disease (GBD) database includes data from 195 countries and territories by age and gender from 1990 to 2017 and is visualized in the form of charts and graphs.

The mortality and CoD (cause of data) database in GBD2017 contains seven types of data sources: vital registration, verbal autopsy, cancer registry, police records, sibling history, surveillance, and survey/census. Countries with complete vital registration systems are considered to be high‐quality. At the national level, these data reports are mainly from the civil registry, local health authority, local police authority, and local administration, etc, and departments such as the Central Statistical Office and the Ministry of Health are responsible for the final death data. For countries with incomplete vital registration systems, vital statistics for causes of death may be supplemented with other data types to provide cause‐specific estimates. Data on cancer incidence were sought from individual population‐based cancer registries as well as from databases that include multiple registries, for example, “Cancer Incidence in Five Continents” (CI5), NORDCAN, or EUREG. The number of cases in cancer registries is mainly from data sources such as local hospitals, community health centers and medical insurance, which are of high quality. The database has set uniform standards to facilitate the collection and collation of data from these cancer registries. Therefore, we assume that the registry has clearly distinguished esophageal cancer from other similar diseases(eg extended gastric cancer) when the incidence and death are reported, or that it has been verified in the standardization of database data inclusion.

Then, according to the social‐demographic index (SDI), these 195 countries and regions are divided into five regions, including low, low‐middle, middle, high‐middle and high.^27^, ^28^ The SDI is a comprehensive indicator for evaluating developmental conditions that are strongly correlated with health outcomes. In short, it is the geometric mean of the total fertility under 25 years old (TFU25), the average education level of people aged 15 and over (EDU15 +)and the lag distributed income per capita (LDI), with a value range of 0 to 1.^29^, ^30^ Moreover, according to the geographic location, the world was divided into 21 regions, eg East Asia.

The ASIR (95% uncertainty interval [UI]) and ASMR (95% UI) of esophageal carcinoma from 195 countries and territories, by gender, country and region, and incidence rate and mortality rate by age groups from 1990 to 2017 were collected from the GHDx query tool. The uncertainty interval is a range of values that reflects the certainty of an estimate. In GBD, every estimate is calculated 1000 times, each time sampling from distributions rather than point estimates for data inputs, data transformations and model choice. The 95th uncertainty interval is determined by the 25th and 975th value of the 1000 values after ordering them from smallest to largest.^31^ The general use of the GBD 2017 database and the estimation methods for disease burden in esophageal cancer have been described in detail in previous studies.^29^, ^32^

Our study used the following parameters to quantify the incidence and mortality trends in esophageal cancer, including age‐standardized incidence rate (ASIR), the age‐standardized mortality rate (ASMR), and estimated annual percentage change (EAPC).^33^ For many purposes, age‐specific comparisons may be the most useful.^34^ However, comparisons of crude age‐specific rates over time and between populations may be very misleading if the underlying age composition differs in the populations being compared. Therefore, given the importance of age standardization, the GBD 2017 have used the nonweighted mean of 2017 age‐specific proportional distributions from the GBD 2017 population estimates for all national locations with a population greater than 5 million people in 2017 to generate a standard population age structure, which is then used as the standard population to calculate the age‐standardized rate (Table S2).^35^

### 2.2 Statistical analysis

Age‐standardized rates in GBD are estimated using the GBD world population age standard, which is calculated using the method of Ahmad et al 2001.^36^ Direct standardization yields a standardized or age‐adjusted rate, which is a weighted average of the age‐specific rates, for each of the populations to be compared. The weights applied to represent the relative age distribution of the arbitrary external population (the standard). This provides, for each population, a single summary rate that reflects the number of events that would have been expected if the populations being compared had identical age distribution. Symbolically, the directly age‐standardized rate for one population is given by the following equation:

$$\text{ASR}\;=\;\frac{\sum_{i=1}^{A}a_{i}w_{i}}{\sum_{i=1}^{A}w_{i}}\times 10,000,$$

where *a_i_* and *w_i_* represent the age‐specific rates and the number of persons (or weight) in the same age subgroup of the chosen reference standard population (where *i* denotes the *i*th age class), respectively.

More importantly, trends in ASR can provide clues for constantly developing risk factors and good surrogates for shifting disease patterns in the population. The EAPC is a good indicator of the ASR trend. By analyzing ASR and EAPC, the effectiveness of current prevention strategies can be established and more targeted strategies can be formulated when necessary.^37^ When calculating the EAPC based on ASR, the calendar year is an independent variable that is used to fit the regression line of the natural logarithm of ASR. The formula was used as:

y=a+bx+ε,

where *y* = ln (ASR) and *x* = calendar year. And there is EAPC = 100 × (exp (*β*) − 1), where *β* is the estimated value of the slope *b*. Then we again apply the above formula to calculate the 95% confidence interval (CI), where the standard error is obtained from the fitted regression line.^38^ If the estimation of EAPC and its lower boundary of 95% CI were both >0, the ASR was considered to be on the rise. On the contrary, if the estimation of EAPC and its upper boundary of 95% CI were both <0, the ASR was considered to be in a downward trend. Otherwise, the ASR was considered to be stable over time.

Finally, considering that the death registration information is more stable and reliable than the incidence registration, the Pearson correlation coefficient between ASMR and EAPC and SDI was calculated. In the correlation analysis, if the Pearson correlation coefficient was <0 and the *P*‐value was <0.05, there was a significant negative correlation between the two variables. All statistics were based on the R programme (Version 3.6.1). A sign of statistical significance is that the *P‐value* is less than 0.05.^39^, ^40^

## 3 RESULTS

### 3.1 Geographic variation of ASIR and ASMR in esophageal cancer

Globally, the number of new cases of esophageal cancer increased from 310.2 thousand (95% UI 300.7, 322.0) in 1990 to 472.5 thousand (95% UI 459.5, 485.3) in 2017. The ASIR decreased from 7.57 (95% UI 7.33, 7.85) per 100 000 in 1990 to 5.90 (95% UI 5.74, 6.06) per 100 000 in 2017, with an EAPC of −1.21 (95% CI −1.41, −1.01) (Table 1). In the same way, the number of deaths from esophageal cancer has increased worldwide from 258.0 thousand (95% UI 240.2, 272.8) in 1990 to 436.0 thousand (95% UI 425.0, 447.6) in 2017. The ASMR decreased from 7.72 (95% UI 7.48, 8.01) per 100 000 in 1990 to 5.48 (95% UI 5.34, 5.63) per 100 000 in 2017, with an EAPC of −1.57 (95% CI −1.79, −1.36) (Table 1).

**TABLE 1:** The age‐standardized incidence rate (ASIR) and age‐standardized mortality rate (ASMR) in 1990 and 2017, and their temporal trends from 1990 to 2017

Characteristics	ASIR/incidence rate (95% UI)		ASMR/death rate (95% UI)		EAPC (95% CI)	
	Per 100 000 in 1990	Per 100 000 in 2017	Per 100 000 in 1990	Per 100 000 in 2017	ASIR/incidence rate（1990‐2017)	ASMR/death rate (1990‐2017)
Overall	7.57 (7.33‐7.85)	5.90 (5.74‐6.06)	7.72 (7.48‐8.01)	5.48 (5.34‐5.63)	−1.21 (−1.41 to −1.01)	−1.57 (−1.79 to −1.36)
Sex						
Male	10.90 (10.54‐11.33)	8.87 (8.55‐9.16)	11.12 (10.74‐11.56)	8.43 (8.15‐8.70)	−1.02 (−1.21 to −0.84))	−1.30 (−1.50 to −1.10)
Female	4.69 (4.47‐4.98)	3.32 (3.17‐3.47)	4.84 (4.61‐5.16)	2.93 (2.81‐3.05)	−1.63 (−1.88 to −1.39)	−2.23 (−2.48 to −1.97)
Age						
15‐49 years	1.36 (1.30‐1.44)	1.04 (1.01‐1.08)	1.11 (1.06‐1.17)	0.75 (0.73‐0.77)	−1.74 (−2.14 to −1.35)	−2.25 (−2.67 to −1.84)
50‐69 years	24.45 (23.66‐25.35)	17.79 (17.28‐18.31)	23.45 (22.67‐24.33)	15.55 (15.13‐16.00)	−1.47 (−1.66 to −1.29)	−1.87 (−2.06 to −1.68)
70 + years	51.62 (49.94‐53.82)	45.58 (44.13‐47.03)	58.90 (56.95‐61.41)	46.60 (45.57‐47.94)	−0.76 (−0.98 to −0.53)	−1.16 (−1.40 to −0.93)
Socio‐demographic index						
Low	5.36 (4.93‐5.78))	4.29 (4.04‐4.57)	5.60 (5.16‐6.05)	4.54 (4.28‐4.84)	−0.99 (−1.17 to −0.83)	−0.95 (−1.12 to −0.77)
Low‐middle	5.18 (4.86‐5.51)	4.42 (4.21‐4.67)	5.40 (5.05‐5.75)	4.63 (4.41‐4.88)	−0.70 (−0.78 to −0.62)	−0.68 (−0.76 to −0.60)
Middle	11.46 (10.96‐12.05)	6.89 (6.55‐7.26)	12.06 (11.52‐12.68)	6.79 (6.45‐7.13)	−2.18 (−2.44 to −1.91)	−2.42 (−2.71 to −2.14)
High‐middle	10.15 (9.69‐10.64)	8.10 (7.62‐8.59)	10.60 (10.12‐11.11)	7.22 (6.84‐7.63)	−1.24 (−1.51 to −0.96)	−1.86 (−2.16 to −1.56)
High	4.42 (4.38‐4.48)	4.30 (4.18‐4.42)	4.14 (4.10‐4.18)	3.50 (3.42‐3.58)	−0.27 (−0.39 to −0.14)	−0.78 (−0.87 to −0.69)
Region						
High‐income Asia Pacific	5.97 (5.87‐6.06)	5.15 (4.87‐5.41)	4.60 (4.54‐4.67)	3.28 (3.14‐3.42)	−0.69 (−0.90 to −0.48)	−1.42 (−1.59 to −1.26)
Central Asia	12.62 (12.32‐12.93)	5.67 (5.40‐5.93)	13.28 (12.96‐13.62)	5.99 (5.71‐6.27)	−3.28 (−3.51 to −3.06)	−3.28 (−3.51 to −3.05)
East Asia	18.92 (18.09‐20.03)	12.09 (11.51‐12.67)	20.03 (19.14‐21.19)	11.09 (10.59‐11.59)	−2.01 (−2.31 to −1.70)	−2.54 (−2.87 to −2.21)
South Asia	4.48 (4.20‐4.76)	3.93 (3.71‐4.17)	4.65 (4.35‐4.95)	4.11 (3.88‐4.36)	−0.69 (−0.83 to −0.55)	−0.66 (−0.80 to −0.53)
Southeast Asia	3.06 (2.74‐3.32)	2.51 (2.36‐2.69)	3.22 (2.88‐3.50)	2.60 (2.45‐2.79)	−0.81 (−0.85 to −0.76)	−0.86 (−0.92 to −0.81)
Australasia	4.51 (4.38‐4.65)	4.45 (4.03‐4.90)	4.04 (3.94‐4.15)	3.77 (3.42‐4.15)	−0.23 (−0.35 to −0.10)	−0.43 (−0.51 to −0.34)
Caribbean	3.61 (3.47‐3.80)	3.29 (3.02‐3.60)	3.85 (3.699‐4.04)	3.40 (3.13‐3.72)	−0.33 (−0.64 to −0.02)	−0.46 (−0.77 to −0.15)
Central Europe	2.94 (2.89‐2.99)	2.68 (2.58‐2.79)	2.99 (2.95‐3.05)	2.73 (2.63‐2.83)	−0.41 (−0.52 to −0.29)	−0.42 (−0.51 to −0.34)
Eastern Europe	3.99 (3.87‐4.12)	3.46 (3.36‐3.58)	3.90 (3.81‐3.98)	2.91 (2.84‐2.995)	−0.89 (−1.18 to −0.60)	−1.51 (−1.78 to −1.24)
Western Europe	4.22 (4.16‐4.29)	3.99 (3.82‐4.18)	4.17 (4.12‐4.23)	3.41 (3.28‐3.54)	−0.35 (−0.43 to −0.27)	−0.89 (−0.96 to −0.82)
Andean Latin America	1.96 (1.86‐2.08)	1.31 (1.18‐1.43)	2.13 (2.01‐2.26)	1.43 (1.29‐1.57)	−1.65 (−1.75 to −1.54)	−1.62 (−1.73 to −1.52)
Central Latin America	2.05 (2.01‐2.09)	1.38 (1.31‐1.44)	2.22 (2.19‐2.26)	1.48 (1.41‐1.54)	−1.76 (−1.90 to −1.61)	−1.81 (−1.95 to −1.66)
Southern Latin America	6.79 (6.62‐6.96)	4.01 (3.72‐4.41)	7.32 (7.14‐7.497)	4.31 (3.99‐4.71)	−2.19 (−2.31 to −2.06)	−2.20 (−2.32 to −2.07)
Tropical Latin America	5.68 (5.57‐5.80)	4.65 (4.53‐4.76)	5.95 (5.84‐6.07)	4.81 (4.70‐4.92)	−0.84 (−0.91 to −0.78)	−0.87 (−0.93 to −0.81)
North Africa and Middle East	2.70 (2.43‐2.98)	2.18 (2.04‐2.35)	2.81 (2.53‐3.12)	2.29 (2.14‐2.48)	−0.83 (−0.95 to −0.71)	−0.77 (−0.90 to −0.65)
High‐income North America	3.61 (3.57‐3.67)	3.88 (3.77‐4.00)	3.49 (3.45‐3.54)	3.47 (3.37‐3.56)	0.04 (−0.13 to 0.22)	−0.23 (−0.36 to −0.10)
Oceania	2.26 (1.96‐2.61)	2.21 (1.91‐2.54)	2.34 (2.03‐2.71)	2.31 (2.00‐2.64)	−0.03 (−0.07 to 0.01)	0.002 (−0.04 to 0.04)
Central Sub‐Saharan Africa	10.38 (9.03‐11.899)	7.26 (6.17‐8.51)	10.90 (9.46‐12.49)	7.67 (6.54‐9.01)	−1.62 (−1.79 to −1.44)	−1.58 (−1.75 to −1.41)
Eastern Sub‐Saharan Africa	10.74 (9.799‐11.78)	7.80 (7.20‐8.57)	11.41 (10.45‐12.49)	8.35 (7.72‐9.17)	−1.45 (−1.58 to −1.33)	−1.41 (−1.53 to −1.29)
Southern Sub‐Saharan Africa	12.31 (11.33‐13.37)	9.96 (9.47‐10.42)	12.68 (11.68‐13.77)	10.53 (10.02‐11.02)	−1.26 (−2.07 to −0.44)	−1.13 (−1.93 to −0.33)
Western Sub‐Saharan Africa	2.93 (2.57‐3.41)	3.98 (3.48‐4.69)	3.09 (2.71‐3.59)	4.24 (3.71‐4.999)	1.43 (1.32 to 1.55)	1.48 (1.37 to 1.60)

Abbreviations: ASIR, age‐standardized incidence rate; ASMR, age‐standardized mortality rate; EAPC, estimated annual percentage change; SDI, socio‐demographic index; CI, confidence interval; UI, uncertainty interval.

**FIGURE 1:**
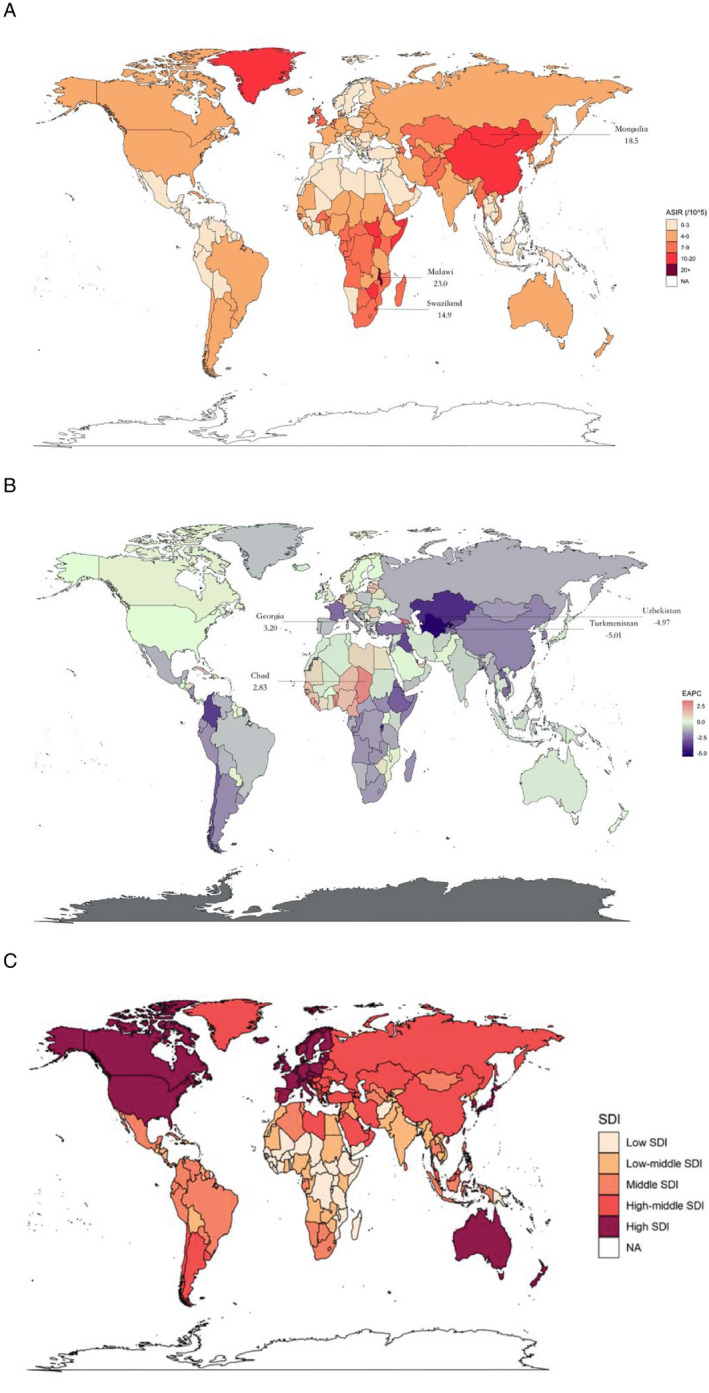
The global disease burden of esophageal cancer for both sexes and all age groups in 195 countries and territories. (A) The ASIR of esophageal cancer in 2017. (B) The EAPC of esophageal cancer ASIR from 1990 to 2017. (C) 5 SDI regions according to socio‐demographic index. ASIR, age‐standardized rate; EAPC, estimated annual percentage change; SDI, socio‐demographic index

**FIGURE 2:**
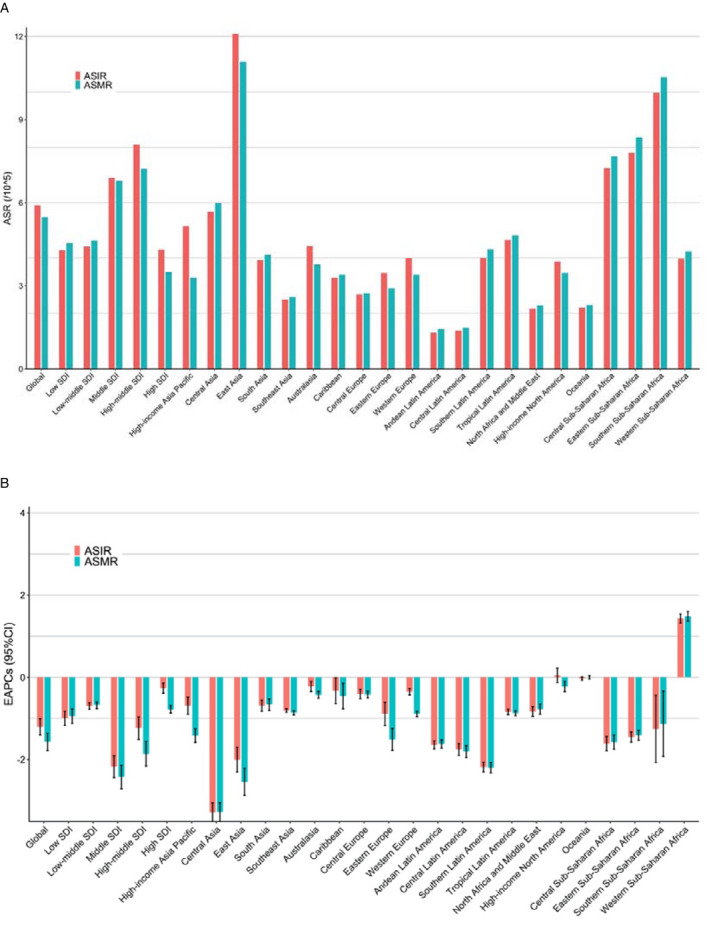
The ASR and EAPCs of esophageal cancer at global, regional, and national levels. (A). The ASIR and ASMR in 2017 for both sexes and all age groups. (B) The EAPCs of ASIR and ASMR from 1990 to 2017 for both sexes and all age groups. ASIR, age‐standardized incidence rate; ASMR, age‐standardized mortality rate; EAPC, estimated annual percentage change; SDI, socio‐demographic index

The ASIR of esophageal cancer varies considerably across the world, with the highest ASIR observed in Malawi (23.0 per 100 000 in 2017), followed by Mongolia and Swaziland (Figure 1A). Similarly, Malawi has the highest ASMR (23.8 per 100 000 in 2017) (Figure S1). In 21 geographic regions, the highest ASIR (12.1 per 100 000 in 2017) and ASMR (11.1 per 100 000 in 2017) existed in East Asia, followed by Southern Sub‐Saharan Africa (Figure 2A). In five SDI regions (Figure 1C), the ASIR (8.1 per 100 000 in 2017) and ASMR (7.2 per 100 000 in 2017) are highest in the high‐middle SDI region (Figure 2A), followed by the middle SDI region.

### 3.2 Temporal variation of ASIR and ASMR in esophageal cancer

In the past nearly three decades, the ASIR and ASMR of esophageal cancer have significantly declined in most countries and territories. The largest decrease was observed in Bahrain (EAPC_(ASIR)_ = −5.31, 95% CI −5.85, −4.76; EAPC_(ASMR)_ = −5.34, 95% CI −5.89, −4.80), followed by Turkmenistan and Uzbekistan (Figure 1B and Table S1). In 21 geographic regions, the largest decline was observed in Central Asia (EAPC_(ASIR)_ = −3.28, 95% CI −3.51, −3.06; EAPC_(ASMR)_ = −3.28, 95% CI −3.51, −3.05), followed by East Asia. In the five SDI regions, the middle SDI region had the highest decreasing trend (EAPC_(ASIR)_ = −2.18, 95% CI −2.44, −1.91; EAPC_(ASMR)_ = −2.42, 95% CI −2.71, −2.14).

However, there are also a few countries and territories that have been observed with significant increasing trends. The largest increase was observed in Georgia (EAPC_(ASIR)_ = 3.20, 95% CI 2.47, 3.94; EAPC_(ASMR)_ = 3.22, 95% CI 2.51, 3.95), followed by Chad and Sao Tome and Principe (Table S1). The only region where ASIR and ASMR increased was Western Sub‐Saharan Africa (EAPC_(ASIR)_ = 1.43, 95% CI 1.32, 1.55; EAPC_(ASMR)_ = 1.48, 95% CI 1.37, 1.60) (Figure 2B).

**FIGURE 3:**
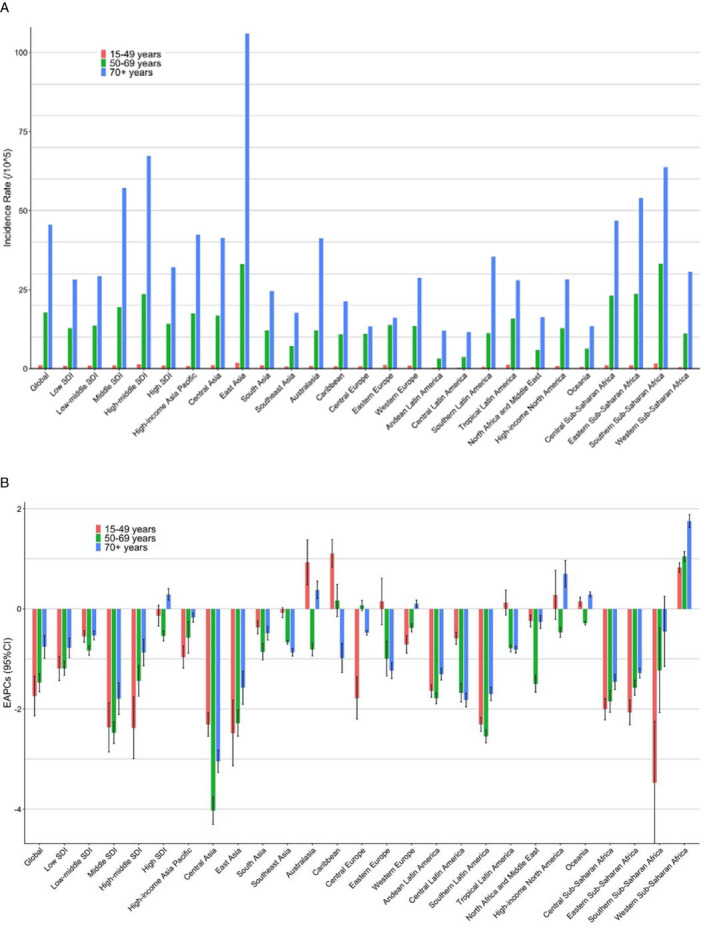
The incidence and EAPCs of esophageal cancer in both sexes and all age groups at global, regional, and national levels. (A) The incidence rate in all age groups in 2017. (B) The EAPCs of incidence rate in all age groups from 1990 to 2017. ASIR, age‐standardized incidence rate;EAPC, estimated annual percentage change; SDI, socio‐demographic index

Overall, the ASIR and ASMR of esophageal cancer in the five SDI regions showed a downward trend (Figure 2B). Further age stratification was performed on the five SDI regions. As shown in Figure 3B, the ASIR and ASMR have increased in the 70+ years age group in the high‐SDI region.

### 3.3 Gender‐specific and age‐specific rates in esophageal cancer

The ASIR and ASMR of esophageal cancer in men were always significantly higher than those in women (Figure S3). Overall, the ASIR in men decreased from 10.90 per 100 000 (95% UI 10.54, 11.33) in 1990 to 8.87 per 100 000 (95% UI 8.55, 9.16) in 2017, with an EAPC of −1.02 (95% CI −1.21, −0.84). The ASIR in women decreased from 4.69 (95% UI 4.47, 4.98) per 100 000 in 1990 to 3.32 (95% UI 3.17, 3.47) per 100 000 in 2017, with an EAPC of −1.63 (95% CI −1.88, −1.39, Figure S2 and Table 1).

We stratified the population according to age, divided into 15‐49 years, 50‐69 years, and 70+ years age groups (because the incidence and mortality rate of esophageal cancer in the population under 15 years is 0, it is not included in this study). The incidence and mortality rate of the 70+ years age group was the highest (Figure 3A, Figures S3, and S4). Age‐stratified EAPC results showed that the morbidity and mortality of esophageal cancer showed a decreasing trend in most regions. And the results also showed that in addition to Western Sub‐Saharan Africa, which had an increasing trend in all age groups, the morbidity and mortality of 15‐49 years age group in Australasia, Caribbean, and Oceania and of 70+ years age group in High‐Income North America, Oceania and high‐SDI regions presented an increasing trend (Figure 3B and Figure S3).

### 3.4 The correlation between ASMR and both EAPC and SDI

**FIGURE 4:**
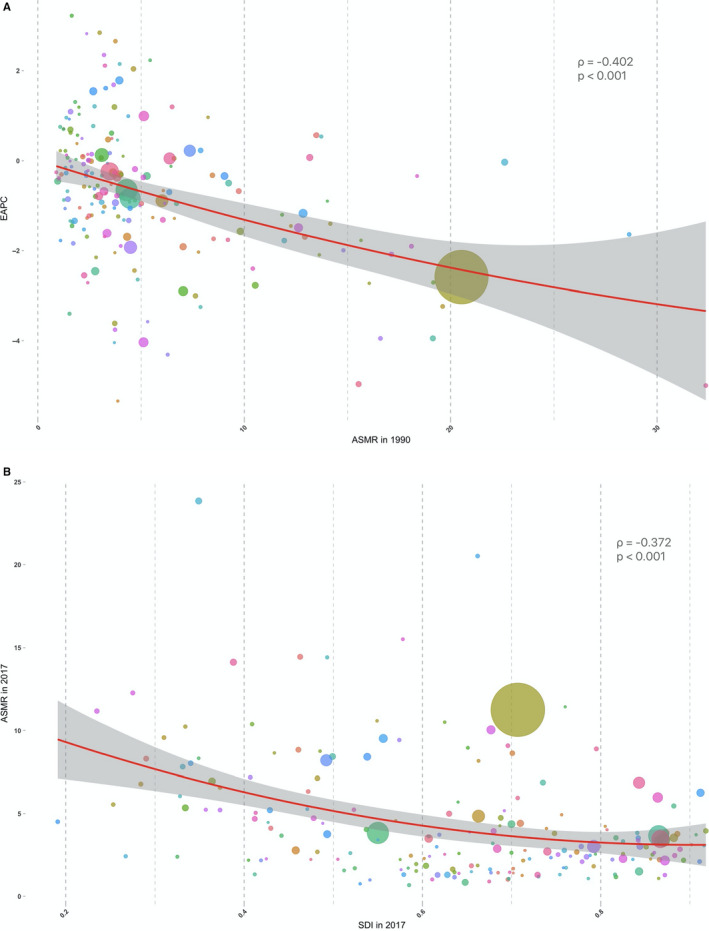
The correlation between ASMR and both EAPC and SDI. (A) The correlation betweenASMRin 1990 and EAPC from 1990 to 2017. (B) The correlation between ASMR in 2017 and SDI in 2017. The circles represent 195 countries and territories. The size of the circle is the current number of cases. The ρ indices and *P* values presented in (A) and (B) were derived from the Pearson correlation analysis. ASMR, age‐standardized mortality rate; EAPC, estimated annual percentage change; SDI, socio‐demographic index

As shown in Figure 4, there were significant negative correlations between ASMR in 1990 and EAPC from 1990 to 2017 (*ρ* = −0.402, *P* < .001) and between ASMR and SDI in 2017 (ρ = −0.372, *P* < .001, Figure 4). The ASMR of esophageal cancer in 1990 reflects the disease reservoir at baseline. When EAPC < 0, EAPC decreases with the increase in ASMR, that is, the higher the baseline ASMR level, the greater the downward trend; when EAPC > 0, EAPC increases with the decrease in ASMR, that is, the lower the baseline ASMR level, the greater the upward trend. The SDI is a comprehensive indicator for evaluating developmental conditions that are strongly correlated with health outcomes. That is, as the level of social population development increases, the ASMR decrease.

## 4 DISCUSSION

This study is the first systematic overview and report on the global burden of esophageal cancer and its morbidity and mortality trends, and it has identified high‐risk populations through gender and age stratification. The principal findings were as follows: (a) The geographical variation of morbidity and mortality in esophageal cancer is large, and the ASIR and ASMR in the highest‐burden regions are 35 times higher than those in the lowest regions. (b) The temporal variation of ASIR and ASMR in esophageal cancer is substantial, and over the past thirty years, the ASIR and ASMR of esophageal cancer in a majority of countries and territories showed a decreasing trend, but an increasing trend existed in Western Sub‐Saharan Africa. (c) The ASIR and ASMR in five SDI regions showed high variation, with higher ASIR and ASMR in middle and high‐middle SDI regions, and decreasing trends, but there was an increasing trend in the 70+ years age group in high SDI regions by age stratification. (d) There were both sex and age specificity in esophageal cancer, in which the ASIR and ASMR were higher in men than in women and the incidence and mortality rate were higher in the 70+ years age group than in other age groups. The decreasing trend was higher in men and in the 70+ years age group, but we found that the incidence and mortality rate of the 15‐49 years age group were increased in Australasia, Caribbean, and Oceania and of the 70+ years age group were increased in High‐income North America and Oceania. (e) There were significant negative associations between ASMR at baseline and EAPC and ASMR and SDI in 2017 respectively.

The reasons for the continued decline in esophageal cancer incidence and mortality in most regions of the world are unclear. There are some theories that might explain this trend. In the United States and Western countries, the majority of cases of ESCC were caused by smoking and excessive drinking.^41^ This also reflects that the incidence of esophageal cancer in males is higher than the rate in females.^42^ Chronic gastroesophageal reflux disease (GERD), Barrett's esophageal disease, obesity, and cigarette smoking have been the risk factors for EAC. In recent years, with the improvements in the socio‐economic level and population health awareness, the decline in smoking rates has partially affected the decline in the incidence of esophageal cancer. We also found a consistent finding with previous studies that the incidence of esophageal cancer has increased in the 70+ years age group in the high SDI region (mostly western countries), which may be related to the fact that GERD has become the prominent cause of esophageal cancer.^43^, ^44^, ^45^ In addition, the previous studies have shown that esophageal cancer is associated with a low intake of certain nutrients, which may be an important cause of esophageal cancer in young non‐smokers (15‐49 years).^46^ A general population supplementation trial in Linxian, China found that combined use of Se, vitamin E, and β‐carotene in people under 55 years of age could reduce ESCC mortality.^47^, ^48^ In Malawi, the country with the highest incidence of esophageal cancer, a nationwide survey carried out in 2011 estimated that Se intake was generally lower, which is mainly caused by reduced soil‐to‐crop Se transfers in the typical low pH soils.^49^, ^50^, ^51^ The previous studies also found that African countries with a higher incidence of esophageal cancer tend to have a lower estimated supply of Fe, Mg, Zn, and Se in their diets, which may be related to the rising trend of esophageal cancer incidence and mortality in Western Sub‐Saharan Africa in our study. This upward trend can also be seen in the study of morbidity and mortality from esophageal cancer in Africa.^52^

In conclusion, our study showed that the geographic variation in the global burden of esophageal cancer, trends in morbidity and mortality over time, and gender‐ and age‐specific population distribution.^53^ Finally, we found that the mortality of esophageal cancer was significantly correlated with EAPC and SDI, which are the quantitative indicators of the changing trend of mortality and quantitative indicators of socio‐demographic development level respectively. However, it should be noted that any conclusions from the correlation should be drawn cautiously due to unadjustable confounders.

Our study has numerous strengths, including the identification of high‐risk populations, which can later allow the targeted exploration of the etiology of esophageal cancer in local regions and provide early prevention strategies to reduce its incidence and mortality rate. Our study also has many limitations. First, in some low SDI and low‐middle SDI regions, the coverage and quality of the data can have a significant impact on the results, leading to results that may need to be supported by more reliable data. In the star‐rating system applied by GBD to assess the quality of available data in various countries and territories, the available data quality level for most countries and regions in sub‐Saharan Africa is 0 star (no vital registration or verbal autopsy data available from 1980‐2017) or 1 star (>0%~9% well‐certified), so the trend of increasing esophageal cancer incidence and mortality in this region has yet to be further verified by more data. Especially in Malawi, which not has reliable data source from vital registration, the disease data on incidence and death are merely based on the insufficiently accurate verbal autopsy, and part of also only cover children under 5 or maternal mortality. Secondly, a cancer diagnosis may be underreported and may lead to bias in cancer registration, especially in countries with limited resources.^46^ According to the principles of filtering and standardization of database data sources, disease ICD codes that cannot be clearly classified are considered as garbage codes. Therefore, causes such as “Injuries” or “Cancer” will be included in the major garbage percentage. Then, the incidence and mortality of esophageal cancer are disproportionately underestimated or overestimated. As a result, morbidity and mortality figures may be somewhat biased, especially in poor countries. Although we have systematically summarized the global burden of esophageal cancer, this burden is too broad, and here it is difficult to translate these data into a pointed study of the etiology of esophageal cancer in a small population. However, we can conduct small‐scale etiological studies and even intervention studies through the high‐risk populations pointedout in this study. For example, previous studies focused on eastern China with a high incidence of esophageal cancer, and the results showed that ESCC was significantly associated with a family history of esophageal cancer, alcohol consumption, and poor oral health.^54^, ^55^, ^56^, ^57^ Next, we will further investigate these findings and explore their basic mechanisms, such as the association between poor oral hygiene and changes in oral microbiota. We will further study how genetic susceptibility and/or environmental exposure lead to an increased risk of disease.

## 5 ETHICS STATEMENT

The data used in this study is publicly available, and patient information is based on the summarized data rather than the individual level.

## CONFLICT OF INTEREST

The authors have no conflict of interest with the information presented.

## AUTHOR CONTRIBUTIONS

Jiahui Fan contributed to the data curation, analysis and interpretation of the data, and manuscript writing; Zhenqiu Liu contributed to the study design, methodology development and interpretation of the data; Xianhua Mao,Xin Tong and Tiejun Zhang critically revised the study protocol and manuscript; Chen Suo and Xingdong Chen contributed to the funding acquisition, study design, methodology, manuscript wiring and supervision. All authors approved the final manuscript. Jiahui Fan and Zhenqiu Liu contributed equally to this article. Chen Suo and Xingdong Chen are co‐correspondence of the article.

## Supporting information

Fig S1

Fig S2

Fig S3

Fig S4

Table S1

Table S2

## Data Availability

The datasets that were analyzed in our study can be obtained from an open data source, using the Global Health Data Exchange (GHDx) query tool (http://ghdx.healthdata.org/gbd‐results‐tool) in the Global Burden of Disease database (http://ghdx.healthdata.org/gbd‐2017/code). The datasets analyzed during the current study are available from the corresponding author on reasonable request.
