# Supplementary figures and images for: Global trends in the incidence and mortality of esophageal cancer from 1990 to 2017
Source: Cancer Med. 2020 Aug 4;9(18):e03338. doi: 10.1002/cam4.3338 (PMC7520289; doi:10.1002/cam4.3338)

a.

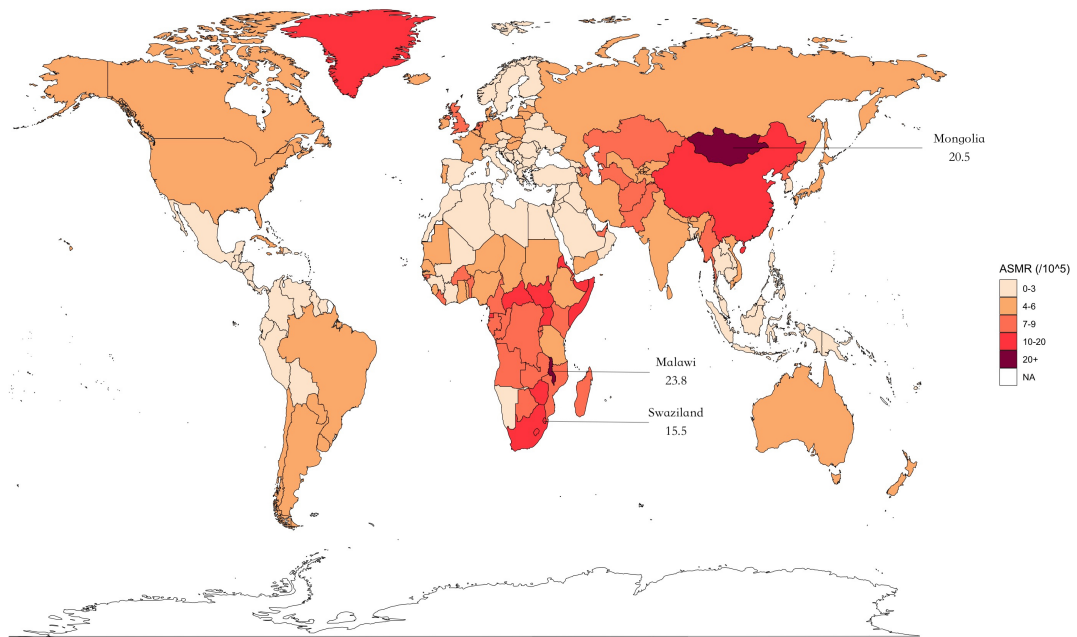

b.

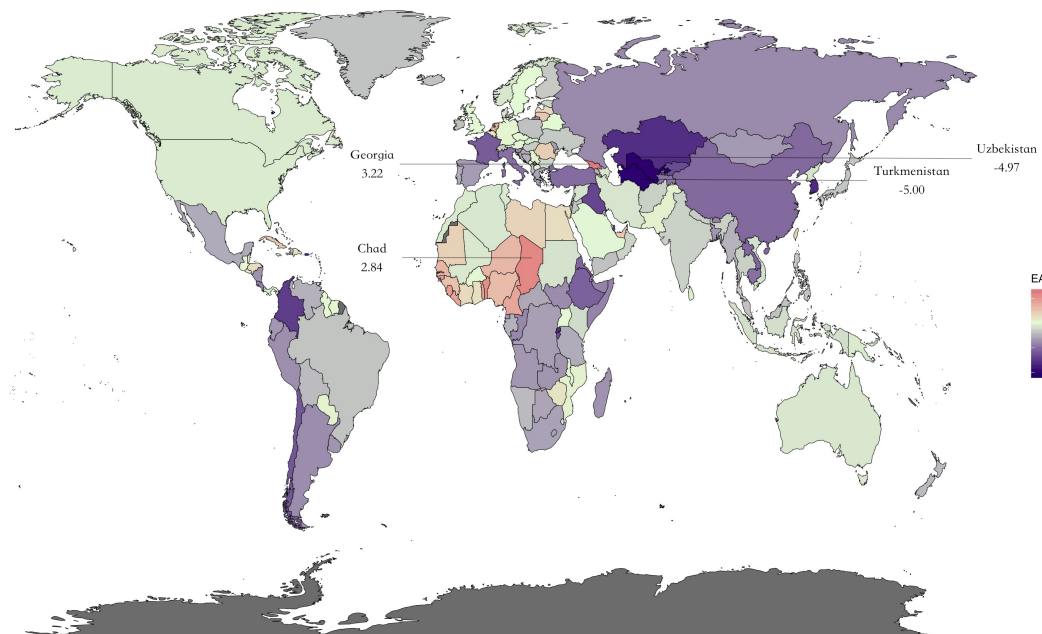

c.

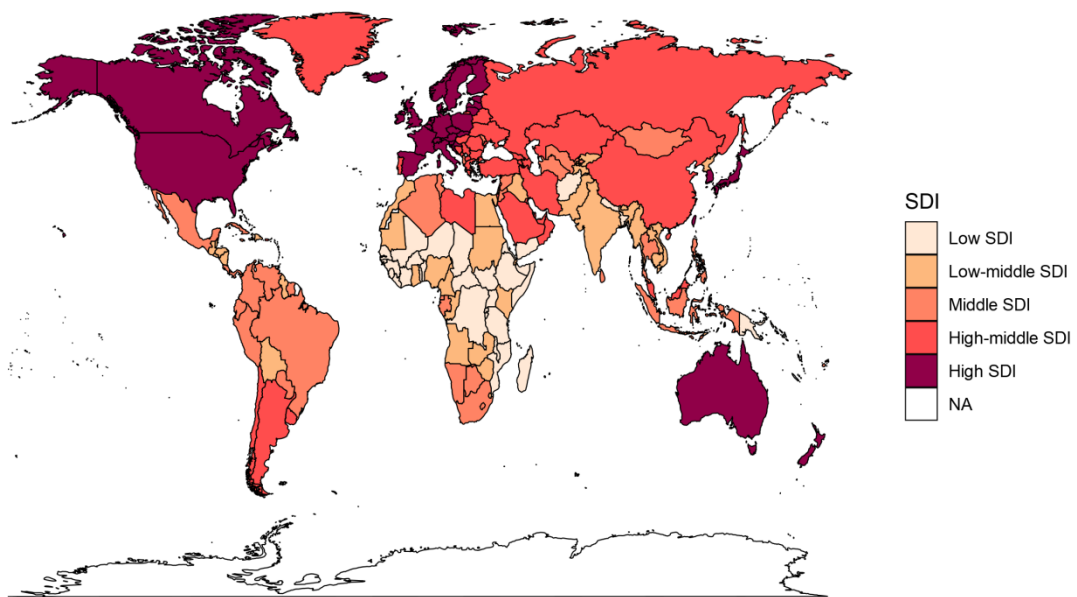

Supplement: Supplementary file 1 — Fig S1 [file CAM4-9-6875-s001.pdf]

a.

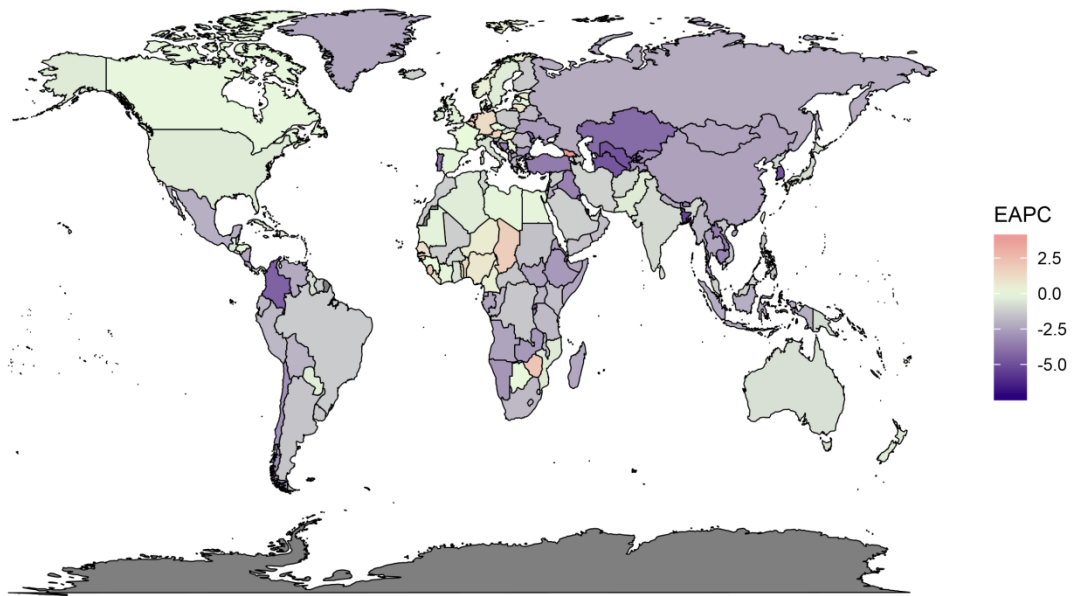

b.

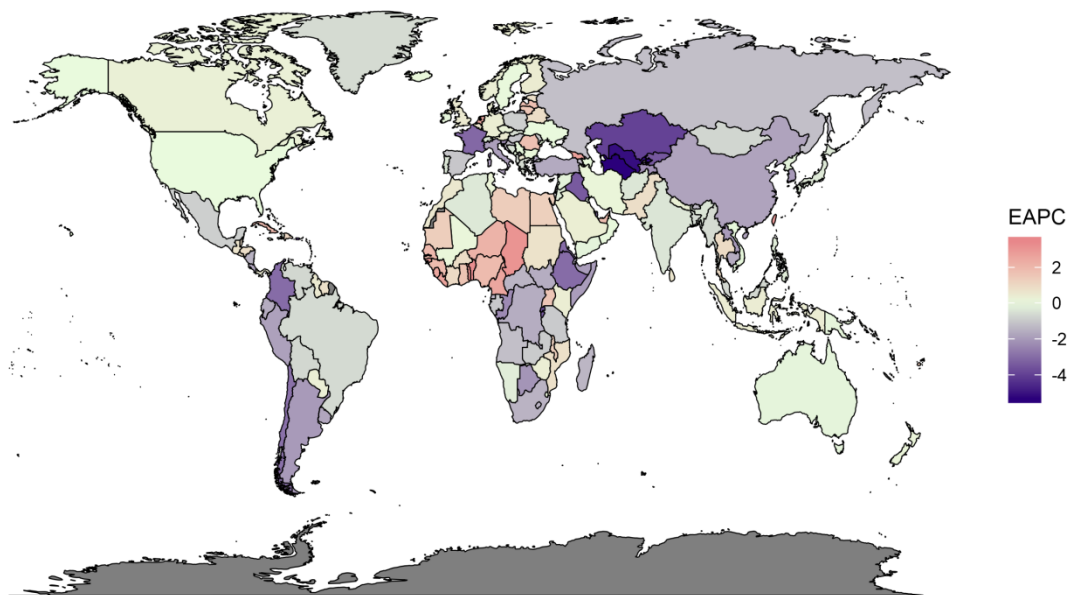

c.

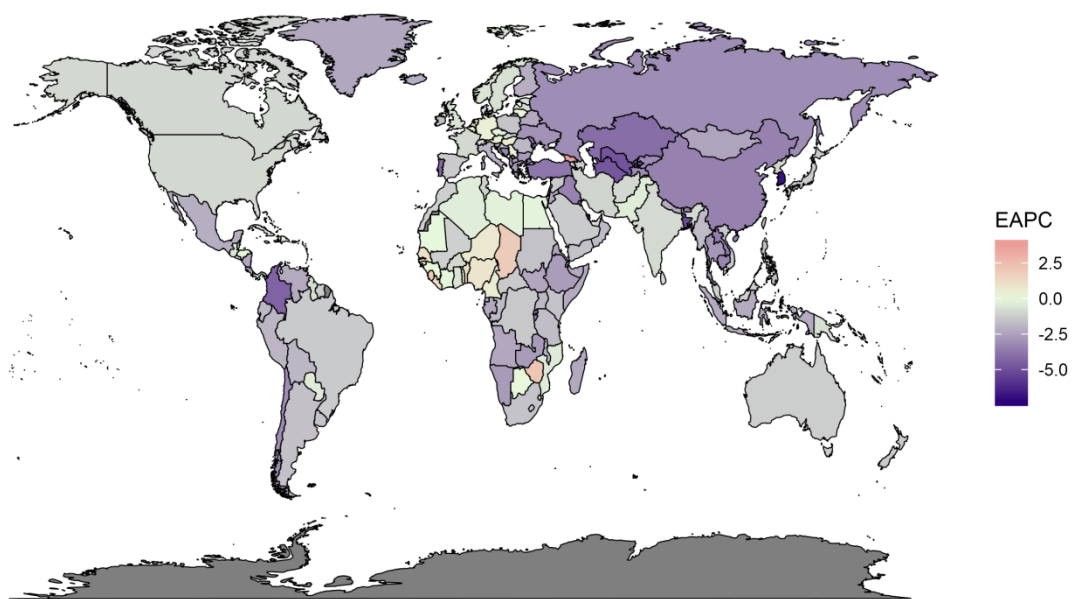

d.

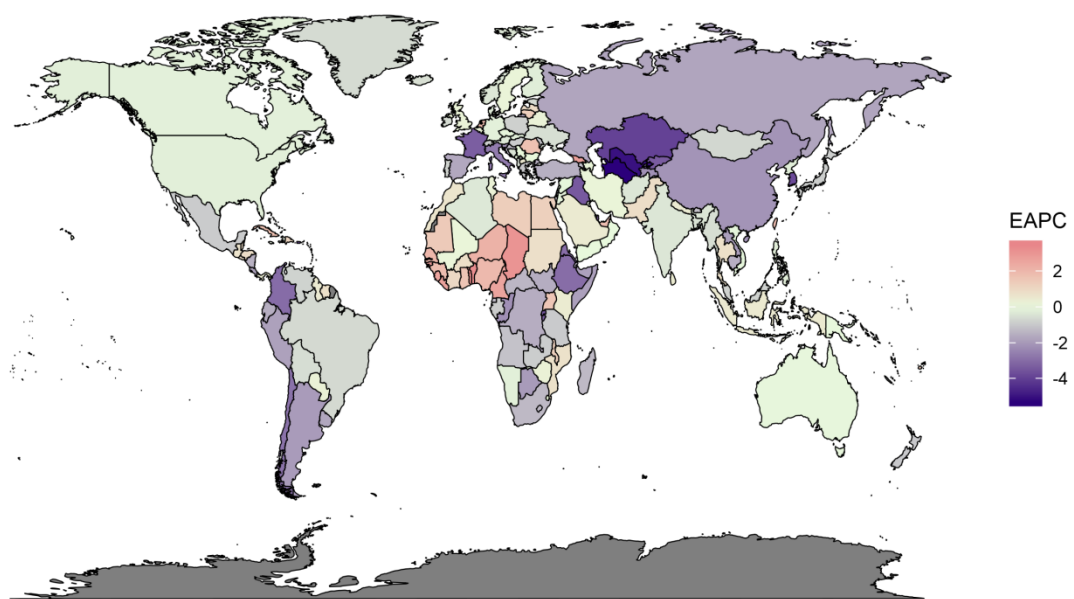

Supplement: Supplementary file 2 — Fig S2 [file CAM4-9-6875-s002.pdf]

a

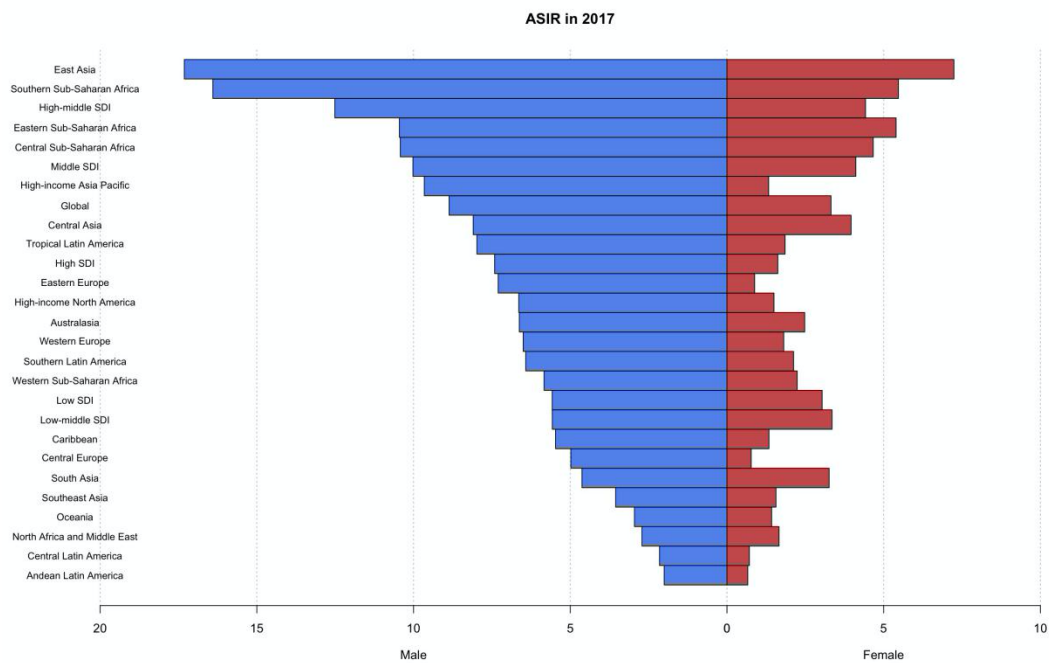

b.

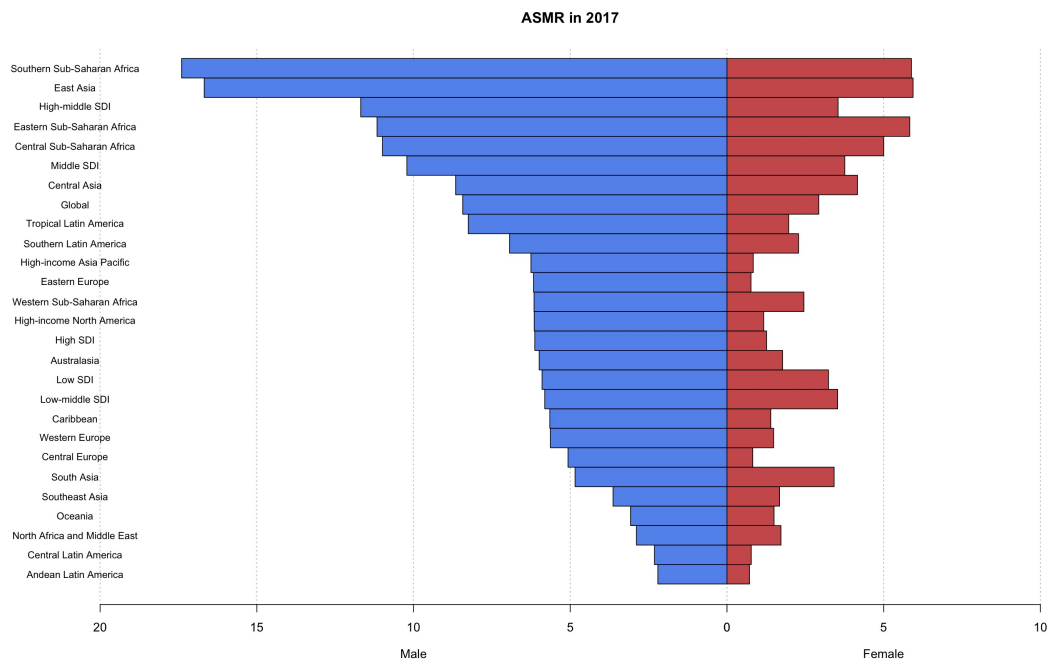

c.

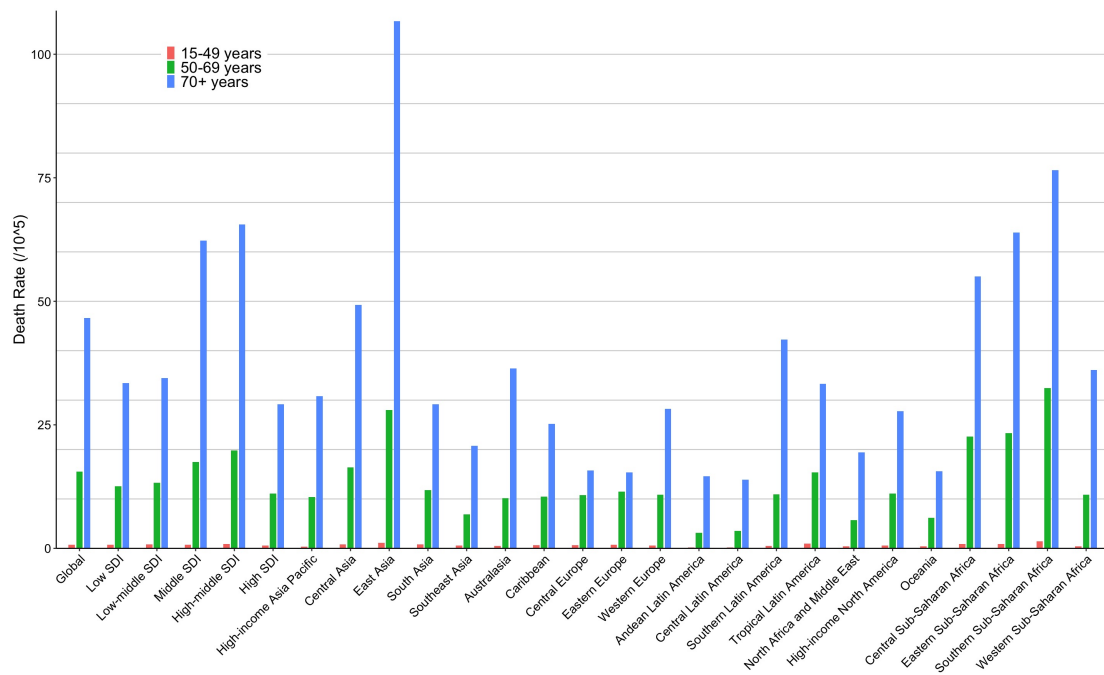

d.

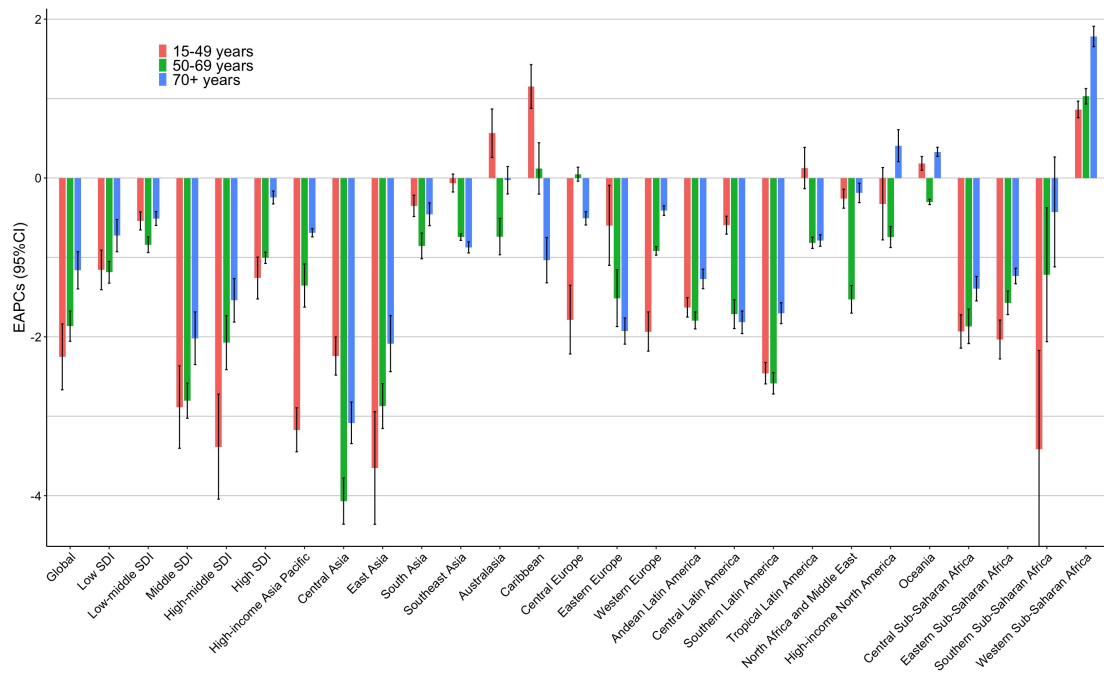

Supplement: Supplementary file 3 — Fig S3 [file CAM4-9-6875-s003.pdf]

a.

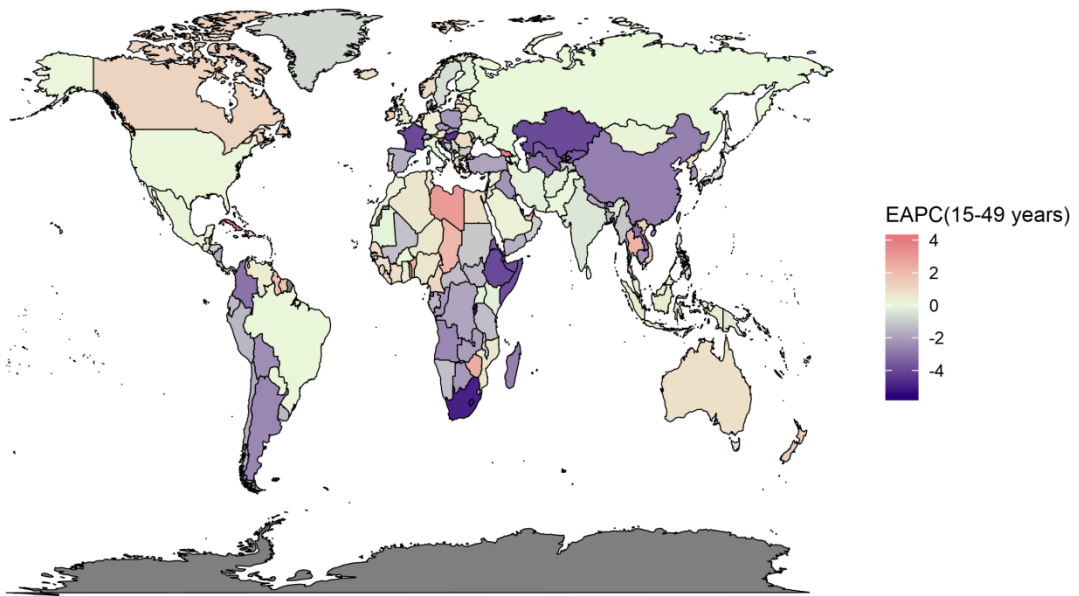

b.

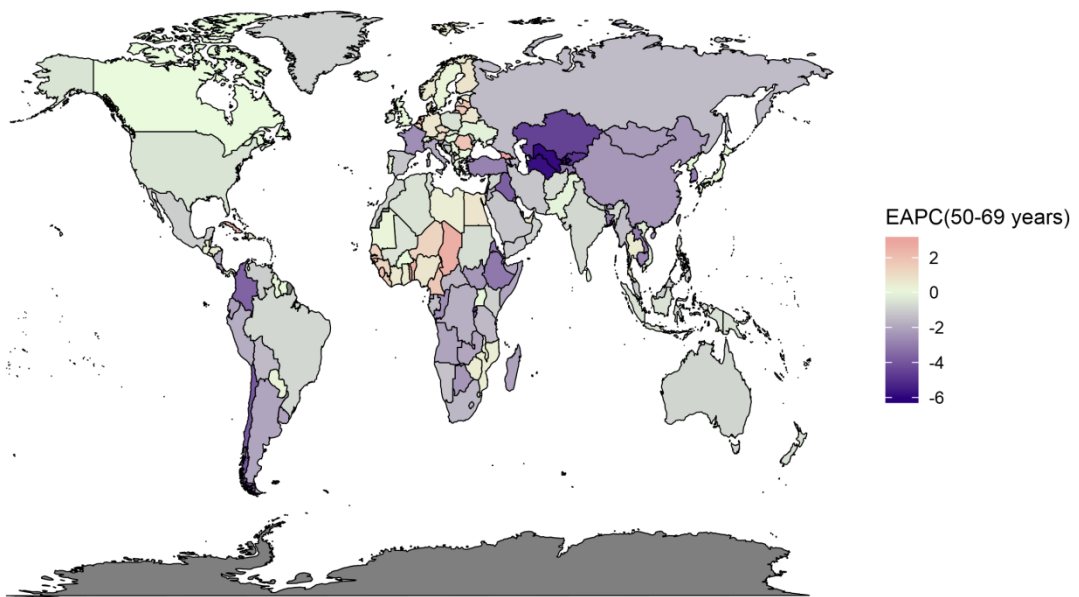

c.

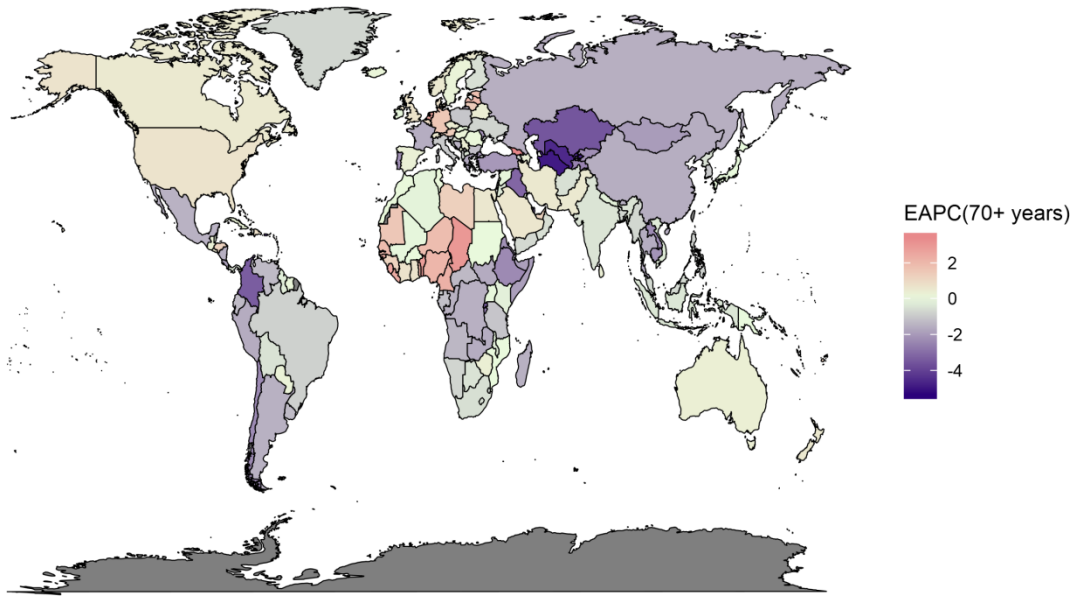

d.

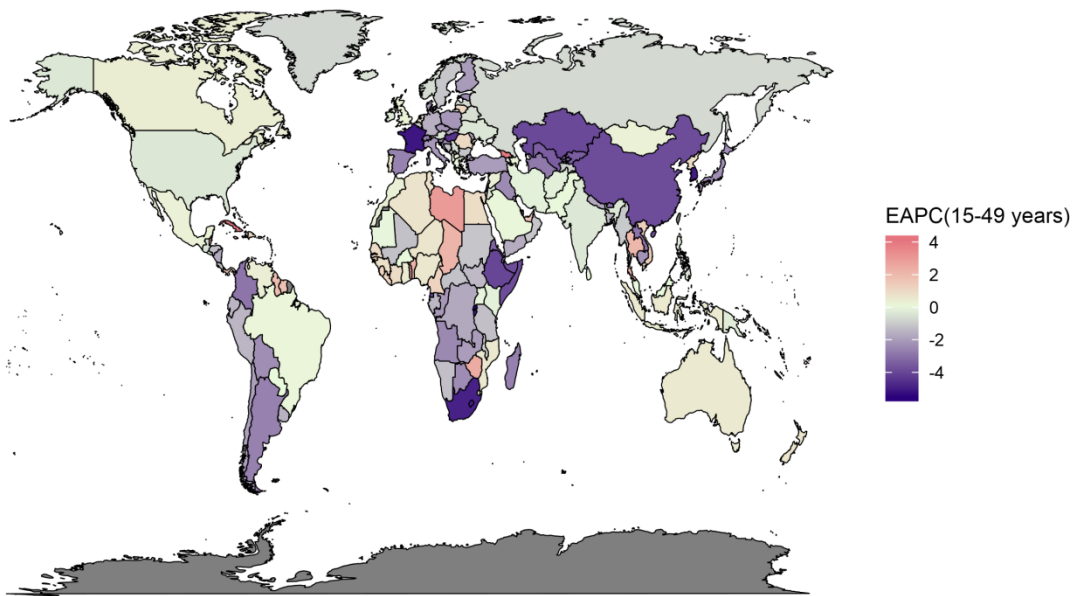

e.

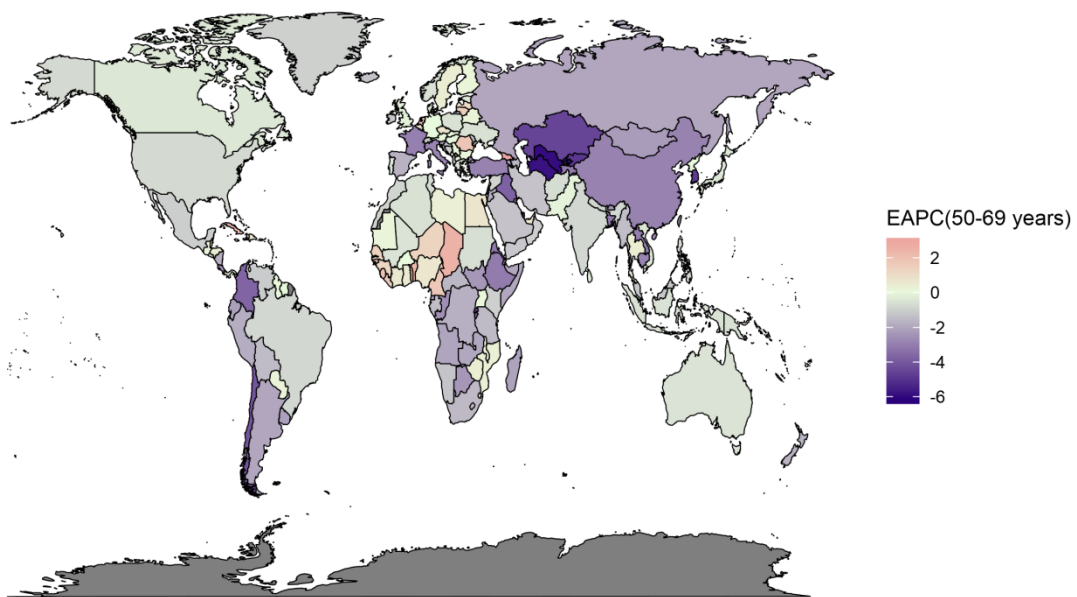

f.

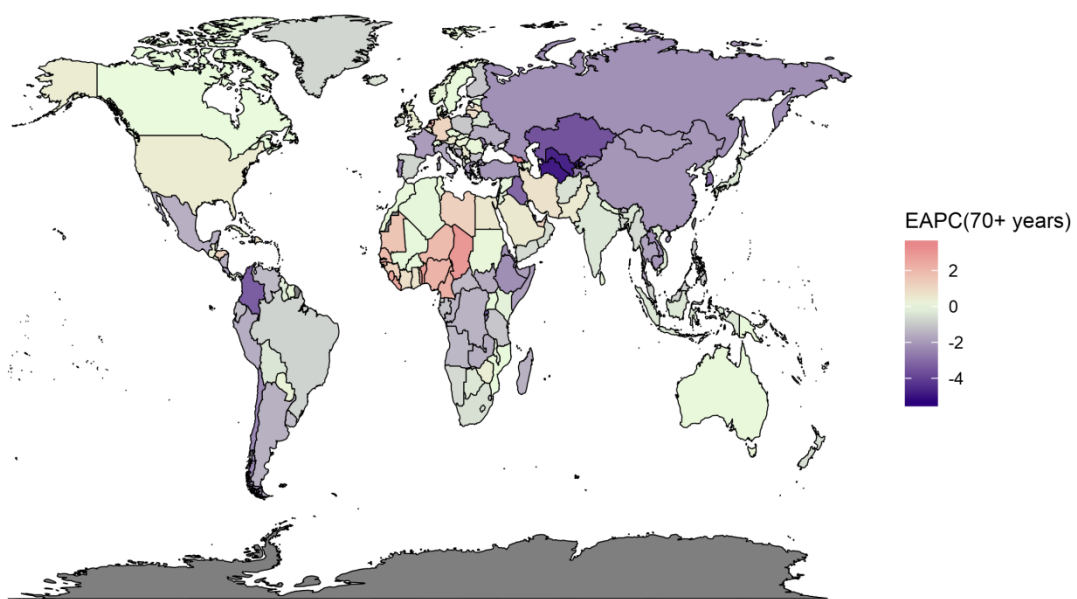

Supplement: Supplementary file 4 — Fig S4 [file CAM4-9-6875-s004.pdf]
