# Supplementary material for: Global trends in the incidence and mortality of esophageal cancer from 1990 to 2017
Source: Cancer Med. 2020 Aug 4;9(18):e03338. doi: 10.1002/cam4.3338 (PMC7520289; doi:10.1002/cam4.3338)
Supplement: Supplementary file 5 — Table S1 [file CAM4-9-6875-s005.pdf]

Region	EAPC (ASIR)	95% CI(lower)	95% CI(upper)	EAPC (ASMR)	95% CI (lower)	95% CI (upper)
Afghanistan	-0.71	-0.82	-0.60	-0.68	-0.79	-0.57
Albania	-1.10	-1.44	-0.77	-1.05	-1.37	-0.72
Algeria	-0.31	-0.40	-0.22	-0.31	-0.40	-0.22
American Samoa	0.74	0.47	1.02	0.72	0.44	0.99
Andorra	0.03	-0.05	0.11	-0.53	-0.58	-0.48
Angola	-1.76	-1.92	-1.60	-1.70	-1.85	-1.54
Antigua and Barbuda	-0.75	-0.91	-0.59	-0.73	-0.88	-0.58
Argentina	-1.91	-2.01	-1.81	-1.91	-2.01	-1.81
Armenia	-1.33	-1.83	-0.82	-1.27	-1.77	-0.77
Australia	-0.28	-0.39	-0.16	-0.30	-0.39	-0.20
Austria	0.96	0.73	1.19	0.09	0.00	0.18
Azerbaijan	-0.42	-0.64	-0.20	-0.32	-0.53	-0.11
Bahamas	-0.69	-0.99	-0.38	-0.66	-0.96	-0.36
Bahrain	-5.31	-5.85	-4.76	-5.34	-5.89	-4.80
Bangladesh	-1.88	-1.99	-1.77	-1.69	-1.83	-1.55
Barbados	-1.24	-1.38	-1.11	-1.26	-1.40	-1.13
Belarus	0.63	0.41	0.84	-0.01	-0.29	0.28
Belgium	0.93	0.66	1.20	0.47	0.20	0.75
Belize	0.48	0.15	0.82	0.38	0.05	0.71
Benin	2.64	2.43	2.86	2.66	2.44	2.87
Bermuda	-1.21	-1.57	-0.85	-1.28	-1.62	-0.94
Bhutan	-2.12	-2.31	-1.93	-2.03	-2.22	-1.85
Bolivia	-1.12	-1.23	-1.02	-1.02	-1.12	-0.92
Bosnia and Herzegovina	-1.21	-1.41	-1.01	-1.21	-1.41	-1.02
Botswana	-1.54	-1.67	-1.41	-1.45	-1.59	-1.31
Brazil	-0.86	-0.92	-0.80	-0.89	-0.95	-0.83
Brunei	-1.61	-1.94	-1.28	-2.05	-2.38	-1.71
Bulgaria	-0.53	-0.92	-0.13	-0.57	-0.96	-0.19
Burkina Faso	0.07	-0.05	0.19	0.05	-0.06	0.17
Burundi	-3.34	-3.65	-3.04	-3.24	-3.53	-2.94
Cambodia	-2.50	-2.67	-2.32	-2.44	-2.62	-2.26
Cameroon	2.00	1.78	2.21	2.04	1.82	2.26
Canada	0.27	0.16	0.38	-0.26	-0.38	-0.15
Cape Verde	1.03	0.80	1.26	0.97	0.75	1.18
Central African Republic	-1.43	-1.56	-1.31	-1.40	-1.52	-1.29
Chad	2.83	2.63	3.04	2.84	2.64	3.05
Chile	-2.96	-3.23	-2.68	-3.00	-3.29	-2.72
China	-2.06	-2.37	-1.75	-2.59	-2.92	-2.25
Colombia	-3.61	-3.80	-3.42	-3.61	-3.81	-3.42
Comoros	-2.81	-3.11	-2.50	-2.73	-3.03	-2.43
Costa Rica	-2.14	-2.59	-1.69	-2.15	-2.59	-1.71
Croatia	-1.22	-1.47	-0.97	-1.24	-1.43	-1.05
Cuba	1.39	1.07	1.71	1.20	0.88	1.52
Cyprus	0.66	0.30	1.01	0.22	-0.16	0.61
Czech Republic	0.36	0.11	0.62	0.07	-0.09	0.22
Democratic Republic of the Cong	-1.59	-1.79	-1.39	-1.57	-1.77	-1.37
Denmark	0.48	0.27	0.68	-0.31	-0.54	-0.08
Djibouti	-2.14	-2.38	-1.91	-2.10	-2.33	-1.86
Dominica	0.14	-0.16	0.45	0.10	-0.20	0.40
Dominican Republic	0.62	0.27	0.97	0.62	0.28	0.97
Ecuador	-1.73	-2.05	-1.40	-1.69	-2.00	-1.38
Egypt	0.71	0.61	0.82	0.69	0.60	0.79
El Salvador	0.18	-0.15	0.50	0.22	-0.09	0.54
Equatorial Guinea	-1.90	-2.20	-1.61	-1.77	-2.05	-1.50
Eritrea	-2.81	-2.99	-2.62	-2.71	-2.89	-2.53
Estonia	0.86	0.42	1.30	-0.55	-0.89	-0.22
Ethiopia	-2.85	-2.96	-2.74	-2.77	-2.88	-2.67
Federated States of Micronesia	-0.61	-0.72	-0.50	-0.58	-0.69	-0.47
Fiji	1.20	1.02	1.38	1.19	1.02	1.36
Finland	-0.05	-0.15	0.04	-0.79	-0.93	-0.66

France	-2.54	-2.61	-2.47	-2.90	-2.97	-2.82
Gabon	-1.23	-1.35	-1.11	-1.20	-1.32	-1.08
Gambia	0.54	0.48	0.59	0.57	0.51	0.63
Georgia	3.20	2.47	3.94	3.22	2.51	3.95
Germany	0.78	0.59	0.97	0.13	0.01	0.25
Ghana	0.62	0.53	0.71	0.62	0.53	0.70
Greece	-0.93	-1.03	-0.83	-1.45	-1.55	-1.35
Greenland	-0.92	-1.05	-0.79	-0.90	-1.03	-0.77
Grenada	-0.05	-0.56	0.45	-0.12	-0.62	0.38
Guam	1.20	0.87	1.54	1.04	0.70	1.37
Guatemala	0.07	-0.31	0.45	0.07	-0.34	0.47
Guinea	1.33	1.22	1.44	1.31	1.20	1.41
Guinea-Bissau	2.20	1.91	2.49	2.23	1.94	2.52
Guyana	0.08	-0.15	0.30	0.03	-0.19	0.25
Haiti	-0.93	-1.12	-0.73	-0.88	-1.07	-0.69
Honduras	0.66	0.51	0.80	0.70	0.54	0.87
Hungary	-0.89	-1.39	-0.40	-0.76	-1.25	-0.27
Iceland	-0.25	-0.42	-0.09	-0.92	-1.13	-0.70
India	-0.68	-0.85	-0.50	-0.65	-0.82	-0.48
Indonesia	-0.46	-0.53	-0.40	-0.45	-0.52	-0.39
Iran	-0.39	-0.59	-0.18	-0.34	-0.54	-0.13
Iraq	-3.40	-3.85	-2.94	-3.40	-3.85	-2.95
Ireland	-0.23	-0.38	-0.08	-0.95	-1.14	-0.76
Israel	-1.14	-1.31	-0.96	-1.37	-1.54	-1.19
Italy	-1.53	-1.61	-1.45	-2.46	-2.59	-2.32
Ivory Coast	0.73	0.39	1.06	0.75	0.42	1.09
Jamaica	-0.27	-0.78	0.24	-0.33	-0.83	0.17
Japan	-0.27	-0.50	-0.04	-0.84	-1.02	-0.65
Jordan	-0.99	-1.14	-0.85	-0.87	-1.01	-0.73
Kazakhstan	-3.97	-4.16	-3.78	-3.95	-4.14	-3.76
Kenya	-0.55	-0.74	-0.36	-0.50	-0.68	-0.32
Kiribati	0.44	0.08	0.79	0.46	0.12	0.81
Kuwait	-1.36	-1.60	-1.12	-1.34	-1.58	-1.10
Kyrgyzstan	-3.35	-3.61	-3.09	-3.25	-3.51	-2.99
Laos	-2.70	-2.87	-2.52	-2.65	-2.82	-2.47
Latvia	1.60	1.26	1.94	0.77	0.44	1.10
Lebanon	-0.44	-0.63	-0.25	-0.68	-0.82	-0.54
Lesotho	0.55	0.26	0.83	0.54	0.26	0.82
Liberia	2.07	1.71	2.44	2.15	1.79	2.51
Libya	0.97	0.84	1.11	0.96	0.82	1.11
Lithuania	1.47	1.13	1.80	1.21	0.88	1.54
Luxembourg	-0.64	-0.75	-0.53	-1.32	-1.43	-1.20
Macedonia	-0.11	-0.26	0.05	-0.15	-0.30	0.00
Madagascar	-1.84	-1.97	-1.72	-1.78	-1.90	-1.66
Malawi	-0.03	-0.45	0.40	-0.03	-0.44	0.38
Malaysia	-0.82	-1.07	-0.56	-0.92	-1.16	-0.69
Maldives	-4.00	-4.23	-3.77	-4.04	-4.27	-3.82
Mali	-0.32	-0.50	-0.14	-0.28	-0.45	-0.11
Malta	-0.55	-0.68	-0.41	-0.96	-1.10	-0.83
Marshall Islands	-0.07	-0.30	0.15	-0.06	-0.28	0.17
Mauritania	0.88	0.64	1.12	0.99	0.76	1.23
Mauritius	-1.06	-1.20	-0.91	-1.14	-1.29	-1.00
Mexico	-1.27	-1.43	-1.10	-1.34	-1.50	-1.18
Moldova	-1.38	-1.79	-0.96	-1.53	-1.92	-1.14
Mongolia	-1.65	-1.99	-1.30	-1.64	-1.97	-1.31
Montenegro	-0.04	-0.16	0.08	0.01	-0.10	0.12
Morocco	-0.32	-0.41	-0.23	-0.27	-0.37	-0.18
Mozambique	0.27	0.17	0.37	0.23	0.14	0.32
Myanmar	-1.24	-1.34	-1.15	-1.17	-1.27	-1.08
Namibia	-1.10	-1.49	-0.71	-1.05	-1.44	-0.67
Nepal	-0.79	-1.40	-0.19	-0.70	-1.30	-0.10
Netherlands	2.47	2.18	2.75	1.78	1.48	2.09
New Zealand	0.04	-0.16	0.24	-1.06	-1.17	-0.95
Nicaragua	-1.77	-1.95	-1.59	-1.78	-1.96	-1.59

Niger	1.55	1.29	1.81	1.61	1.35	1.87
Nigeria	1.46	1.32	1.60	1.54	1.40	1.69
North Korea	-0.30	-0.43	-0.18	-0.34	-0.48	-0.20
Northern Mariana Islands	1.11	0.76	1.45	0.93	0.61	1.24
Norway	0.52	0.34	0.70	-0.33	-0.44	-0.22
Oman	-0.59	-0.74	-0.44	-0.63	-0.78	-0.47
Pakistan	0.17	-0.06	0.40	0.22	0.00	0.44
Palestine	-1.08	-1.32	-0.85	-1.06	-1.30	-0.83
Panama	-0.03	-0.27	0.22	-0.13	-0.37	0.11
Papua New Guinea	-0.31	-0.35	-0.28	-0.25	-0.29	-0.22
Paraguay	0.22	0.07	0.38	0.17	0.02	0.32
Peru	-1.83	-2.10	-1.57	-1.84	-2.11	-1.57
Philippines	-0.84	-1.00	-0.68	-0.86	-1.02	-0.69
Poland	-0.93	-1.01	-0.84	-0.93	-1.02	-0.85
Portugal	-0.94	-1.14	-0.75	-1.33	-1.44	-1.22
Puerto Rico	-4.14	-4.39	-3.89	-4.31	-4.56	-4.06
Qatar	-3.51	-3.77	-3.25	-3.58	-3.84	-3.31
Republic of Congo	-2.09	-2.28	-1.89	-1.99	-2.18	-1.81
Romania	1.14	0.83	1.45	1.09	0.79	1.40
Russia	-1.28	-1.59	-0.97	-1.93	-2.22	-1.63
Rwanda	-4.10	-4.49	-3.70	-3.95	-4.33	-3.57
Saint Lucia	-0.96	-1.28	-0.63	-1.00	-1.32	-0.68
Saint Vincent and the Grenadines	0.55	0.16	0.94	0.50	0.11	0.89
Samoa	-0.08	-0.18	0.01	-0.01	-0.11	0.09
Sao Tome and Principe	2.78	2.55	3.00	2.82	2.60	3.05
Saudi Arabia	-0.11	-0.39	0.17	-0.07	-0.33	0.18
Senegal	1.64	1.37	1.91	1.68	1.42	1.95
Serbia	0.11	-0.04	0.26	0.15	0.00	0.29
Seychelles	-0.91	-1.01	-0.82	-0.95	-1.04	-0.86
Sierra Leone	2.30	2.11	2.50	2.35	2.15	2.55
Singapore	-2.03	-2.20	-1.86	-3.76	-3.91	-3.60
Slovakia	-0.55	-0.90	-0.19	-0.27	-0.50	-0.04
Slovenia	-1.98	-2.12	-1.84	-1.90	-2.03	-1.76
Solomon Islands	-0.27	-0.42	-0.13	-0.22	-0.36	-0.08
Somalia	-2.15	-2.39	-1.92	-2.08	-2.31	-1.86
South Africa	-1.66	-2.57	-0.75	-1.49	-2.38	-0.59
South Korea	-2.22	-2.41	-2.04	-4.03	-4.30	-3.77
South Sudan	-1.93	-2.15	-1.71	-1.90	-2.12	-1.69
Spain	-0.94	-1.04	-0.84	-1.61	-1.71	-1.52
Sri Lanka	-0.06	-0.52	0.40	-0.19	-0.67	0.30
Sudan	-0.43	-0.52	-0.34	-0.37	-0.47	-0.27
Suriname	0.14	-0.07	0.35	0.13	-0.09	0.34
Swaziland	-0.32	-0.80	0.16	-0.34	-0.81	0.13
Sweden	0.01	-0.18	0.20	0.01	-0.11	0.13
Switzerland	-0.24	-0.45	-0.04	-0.78	-0.95	-0.61
Syria	-0.42	-0.64	-0.20	-0.39	-0.60	-0.17
Taiwan	2.24	2.04	2.43	1.00	0.78	1.22
Tajikistan	-2.37	-2.84	-1.91	-2.40	-2.86	-1.94
Tanzania	-1.34	-1.51	-1.16	-1.32	-1.49	-1.15
Thailand	-0.36	-0.58	-0.14	-0.68	-0.89	-0.47
Timor-Leste	-1.74	-1.93	-1.55	-1.69	-1.88	-1.50
Togo	2.09	1.91	2.27	2.11	1.93	2.29
Tonga	0.17	-0.04	0.39	0.14	-0.08	0.36
Trinidad and Tobago	-2.66	-3.05	-2.26	-2.72	-3.11	-2.31
Tunisia	-0.26	-0.40	-0.11	-0.26	-0.39	-0.12
Turkey	-2.54	-3.09	-2.00	-2.55	-3.11	-1.99
Turkmenistan	-5.01	-5.80	-4.21	-5.00	-5.78	-4.22
Uganda	0.06	-0.20	0.31	0.07	-0.17	0.32
UK	0.33	0.13	0.54	0.05	-0.15	0.25
Ukraine	-0.26	-0.61	0.10	-0.79	-1.12	-0.46
United Arab Emirates	1.24	1.02	1.47	1.20	0.97	1.43
Uruguay	-1.74	-1.89	-1.60	-1.76	-1.91	-1.61
USA	0.01	-0.19	0.20	-0.24	-0.38	-0.10
Uzbekistan	-4.97	-5.31	-4.62	-4.97	-5.32	-4.61
Vanuatu	0.05	0.02	0.08	0.11	0.08	0.13
Venezuela	-1.15	-1.53	-0.77	-1.23	-1.62	-0.83
Vietnam	-0.32	-0.40	-0.24	-0.37	-0.46	-0.28
Virgin Islands, U.S.	0.58	0.43	0.73	0.50	0.35	0.65
Yemen	-1.01	-1.10	-0.91	-0.95	-1.05	-0.86
Zambia	-1.77	-2.05	-1.49	-1.74	-2.01	-1.47
Zimbabwe	0.62	-0.03	1.26	0.57	-0.06	1.20
