# Supplementary material for: Global trends in the incidence and mortality of esophageal cancer from 1990 to 2017
Source: Cancer Med. 2020 Aug 4;9(18):e03338. doi: 10.1002/cam4.3338 (PMC7520289; doi:10.1002/cam4.3338)
Supplement: Supplementary file 6 — Table S2 [file CAM4-9-6875-s006.pdf]

| Age Group | Persent of Population | Rounded |
| --- | --- | --- |
| 0-1 | 2.078241644 | 2.08 |
| 1-4 | 8.102445249 | 8.1 |
| 5-9 | 9.677325318 | 9.68 |
| 10-14 | 8.952609678 | 8.95 |
| 15-19 | 8.382858071 | 8.38 |
| 20-24 | 8.01707612 | 8.02 |
| 25-29 | 7.778732811 | 7.78 |
| 30-34 | 7.331586983 | 7.33 |
| 35-39 | 6.775951151 | 6.78 |
| 40-44 | 6.089034035 | 6.09 |
| 45-49 | 5.465811837 | 5.47 |
| 50-54 | 4.873621621 | 4.87 |
| 55-59 | 4.251640217 | 4.25 |
| 60-64 | 3.5961672 | 3.6 |
| 65-69 | 2.914902912 | 2.91 |
| 70-74 | 2.130279933 | 2.13 |
| 75-79 | 1.608333391 | 1.61 |
| 80-84 | 1.078423497 | 1.08 |
| 85-89 | 0.603162091 | 0.6 |
| 90-94 | 0.234261019 | 0.23 |
| 95+ | 0.057535221 | 0.06 |
